# A shell matrix protein of *Pinctada mazatlanica* produces nacre platelets in vitro

**DOI:** 10.1038/s41598-020-77320-7

**Published:** 2020-11-19

**Authors:** Crisalejandra Rivera-Perez, Iliana Alejandra Flores-Sánchez, Josafat Jehu Ojeda Ramírez de Areyano, Delia Irene Rojas Posadas, Norma Y. Hernández-Saavedra

**Affiliations:** 1 CONACYT-Centro de Investigaciones Biológicas del Noroeste (CIBNOR), Avenida Instituto Politécnico Nacional No. 195, Playa Palo de Sta. Rita Sur, Apartado Postal 128, 23096 La Paz, Baja California Sur Mexico; 2 Tecnológico Nacional de México, La Paz, Baja California Sur Mexico; 3 Centro de Investigaciones Biológicas del Noroeste (CIBNOR), Avenida Instituto Politécnico Nacional No. 195, Playa Palo de Sta. Rita Sur, Apartado Postal 128, 23096 La Paz, Baja California Sur Mexico

**Keywords:** Biochemistry, Molecular biology

## Abstract

Nacre is the main component of the pearl oyster shells and it is synthesized by specialized soluble and insoluble shell matrix proteins. Insoluble proteins from the decalcification of the shell are the less studied proteins due to the technical problems to isolate them from the organic matrix. In this study, an insoluble shell matrix protein from *Pinctada mazatlanica*, pearlin (Pmaz-pearlin), was successfully cloned from the mantle tissue, and the native protein isolated from the shell was functionally characterized. The full coding sequence of Pmaz-pearlin mRNA consists of 423 base pairs, which encode to a 16.3 kDa pearlin. Analysis of the deduced amino acid sequence revealed that Pmaz-pearlin contained four acidic regions, an NG repeat domain, and Cys conserved residues, the latter potentially forms four disulfide bridges which might stabilize the protein structure. The isolated protein from the shell is a glycoprotein of ~ 16.74 kDa which can produce aragonite and calcite crystals in vitro. Our results show that Pmaz-pearlin is a well-conserved protein involved in nacre layer growth, which produces calcite crystals in the presence of CaCl_2_, aragonite crystal polymorphs with a hexagonal structure in the presence of MgCl_2_, and needle-like crystal structure polymorphs in the presence of CaCO_3_ The identity of the crystals was confirmed using RAMAN analyses.

## Introduction

The Mollusk shell is composed of aragonite and calcite, crystal polymorphs of calcium carbonate. The outer prismatic layer of the shell is formed by calcite and the inner nacreous layer is composed of aragonite. These structures are made by specialized proteins known as shell matrix proteins (SMPs), which are synthesized by the epithelial cells from the mantle of mollusks^1^ and released into the extrapallial space, between the mantle and the shell, where they perform the crystal nucleation, crystal growth and crystal regulation^2^. The SMPs have been classified according to their solubility after shell decalcification with EDTA or acetic acid solutions as soluble acidic matrix (Asp-rich proteins) and insoluble framework matrix proteins (Gly and Ala-rich proteins), the latter is mostly composed by chitin and silk^3,4^.

Soluble SMPs are known to determine the mineralogical and crystallographic properties of the shell^5^, while insoluble proteins create the necessary microenvironments for crystal growth and supply a surface for specific molecular reorganization, the framework for the shell formation^6^. Several insoluble framework proteins had been described from the nacreous and the prismatic layer e.g. N14/N16/pearlin^4^, Pif80^7^, Pif97^8^, MSI proteins (7, 31 and 60)^9^, Fam20c^10^, N25^11^, nacrein^12^, shematrin^13^, silkmapin^14^. Most of these proteins share specific domains which have been related to functions such as inhibitors of growth and precipitation of calcite^15^ and/or inducers of aragonite nucleation^4^. Proteins such as MSI60 acts as structural support for crystal nucleation and growth due to the self-assembly of the protein forming a fiber-like structure^9^, other structural proteins includes hic31 and hic52, both containing polyglycine blocks of (Gly)_n_ and structure similar to collagen type I, alpha 1 and alpha 2^16–18^. Pearlin is an insoluble SMPs that has drawn attention since is involved in the shell nacre formation and the pearl of oysters. Pearlin belongs to a family protein of low molecular weight, which includes pearlin, N14 and N16 proteins^4,19–21^. They differ only by a few amino acids and exhibit moderately acid and basic isoelectric point^19^. The members of this family have a molecular weight of 13.6 to 16.0 kDa^20–25^, however, N14 can dimerize forming a 28 kDa protein^19^. The amino acid sequences of the members of this family are mainly composed of Gly and Tyr^26^. Also, they exhibit an NG repeat domain which differs in length and has been hypothesized to be involved in the regulation of the crystal growth^4,27,28^. Moreover, they possess four short acidic domains, as well as disulfide bridges at conserved positions^24^.

Isolation, cDNA cloning, and characterization of pearlin proteins have been limited to two oyster species, *Pinctada fucata*^21,29^ and *Pinctada margaritifera*^24^. The pearlin transcript is highly expressed in the dorsal zone of the mantle^21,24^. The resulting protein is a monomeric glycoprotein of 13.6 to 15.0 kDa^21,24^, composed by a high proportion of Gly, Tyr, Cys, Asn, Asp and Arg^21^ and with calcium-binding properties. These proteins contain in their modular structure four acidic rich regions, ten conserved cysteine residues, a putative casein kinase II phosphorylation site (TDDD) and NG domain, the latter differ significantly in length between them, 10 and 35 residues respectively^21,24^. These proteins can induce aragonite crystallization^26^ and establish inside the interlamellar matrix that separates layers^24^, however, in vitro studies shown that high protein concentration (above 10 µg mL^−1^) acts as an inhibitor of the precipitation of calcium carbonate, forming crystals of smaller size^24^. Also, the modulation of the crystal growth has been hypothesized to be due to the presence of cofactors and/or the intrinsic properties of the protein, such as posttranslational modifications (e.g. phosphorylation, glycosylation, etc.)^30^.

Although biomineralization in mollusk has been studied in several species, the basic mechanism responsible for the inner nacre formation remains unknown. Thus, it is important to characterize the proteins related to this structure. The present study describes the isolation and characterization of the pearlin transcript from the mantle and the pearlin native protein from the shell of the pearl oyster *Pinctada mazatlanica,* the latter was functionally characterized. The structural properties of the pearlin were compared to homologs proteins and the role of pearlin in the shell formation is discussed. The results obtained from this study are beneficial for further studies to obtain a comprehensive understanding of the nacre formation during the biomineralization processes.

## Results

### Sequence analysis of cDNA pearlin from the mantle of *P. mazatlanica*

**Figure 1:**
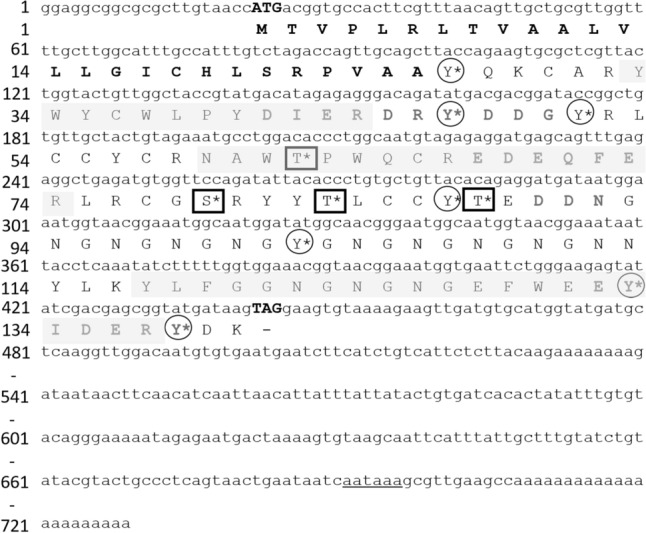
Nucleotide and deduced amino acid sequence in *Pinctada mazatlanica* pearlin cDNA (GenBank: MT746149). The putative signal peptide is indicated in bold letters. Acidic regions, identified by literature, are marked by gray letters. Potential O-glycosylation and N-glycosylation residues are marked by square and circle shapes. Putative phosphorylation sites are marked with an asterisk (*). Amino acid sequences identified by Mass Spectrometry are shaded in gray. The initiation codon (ATG) and the stop codon (TGA) are shown in bold and uppercase. The putative polyadenylation signal (AATAAA) is underlined.

**Figure 2:**
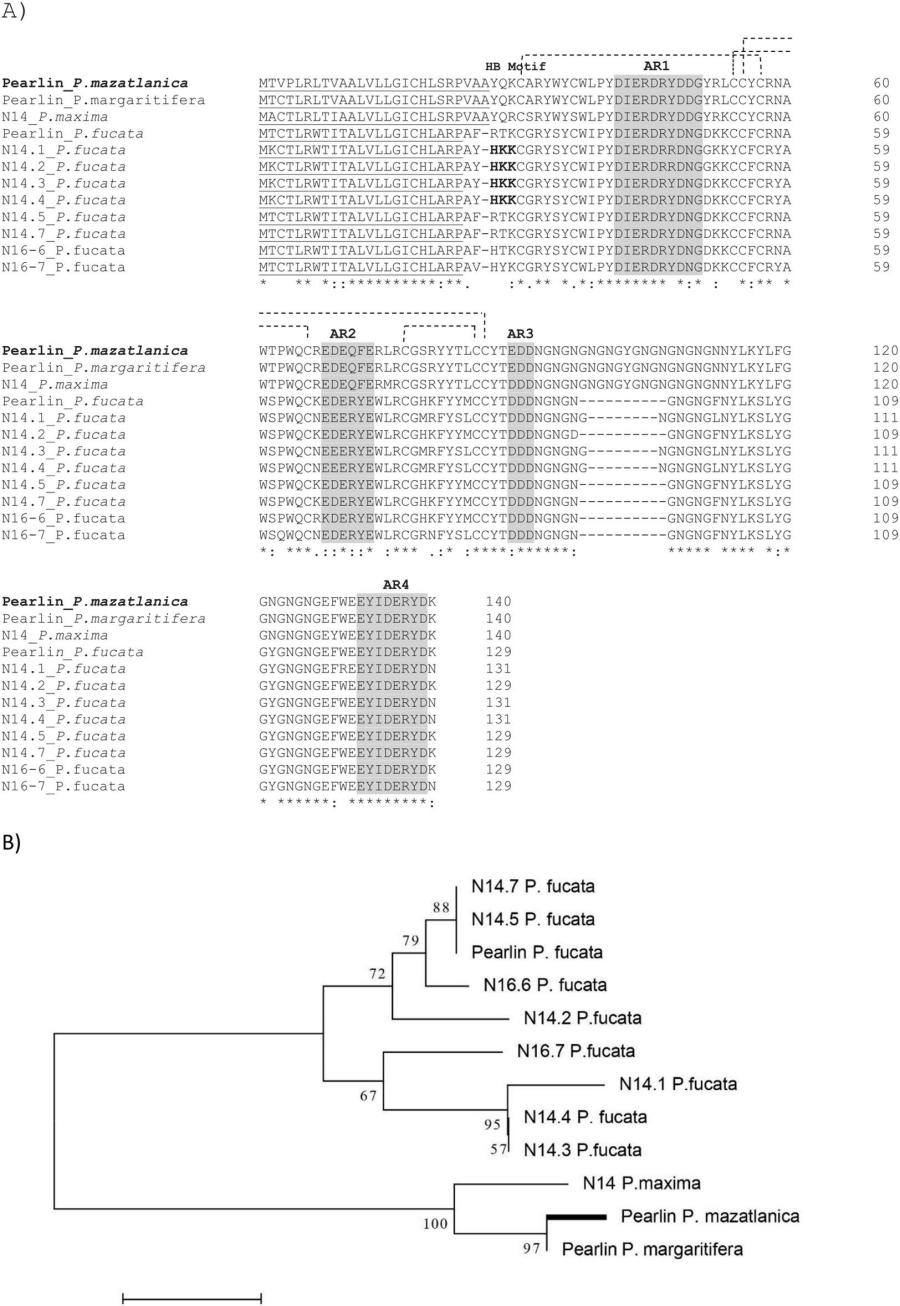
Comparison of the deduced amino acid sequence of pearlin and related proteins from pearl oyster species. (**A**) Alignments were made with Clustal Omega (version 1.2.4). Heparin-binding motif (HB motif) and acidic regions (AR) are indicated. Signal peptide is underlined, and putative disulfide bridges are shown by connected dotted lines between cysteine amino acid residues. (**B**) Neighbor-Joining phylogenetic tree of pearlin from *P. mazatlanica* and related proteins. Protein sequences, pearlin from *P. mazatlanica* (GenBank no. MT746149), *P. margaritifera* (GenBank no. ABG24165.3), *P. fucata* (GenBan no. BAA75626.1), N14 from *P. maxima* (BAA90539.1), and N14.1 (GenBank no. BAA83733.1), N14.2 (GenBank no. BAA83734.1), N14.3 (GenBank no. BAA83735.1), N14.4 (GenBank no. BAA83736.1), N14.5 (GenBank no. BAA83737.1), N14.7 (GenBank no. BAA83739.1), N16-6 (GenBank no. BAN84255.1) and N16-7 (GenBank no. BAN67811) from *P. fucata*. *Asterisk* shows identical or conserved residues in all sequences in the alignment; *colons* are conservative substitutions; *periods* are semi-conservative substitutions.

The full-length Pmaz-pearlin was composed of a 726-bp including 5′-UTR and 3′-UTR (292 bp), and an open reading frame (ORF) of 423 bp encoding 140 amino acids (Fig. 1). The Pmaz-pearlin has a calculated molecular mass of 16.3 kDa and a theoretical pI of 5.16 before any post-translational modification (PTM). Removal of the signal peptide sequence (residues 1–26) resulted in a theoretical molecular weight of 13.6 kDa and a theoretical pI of 4.69. It possesses high proportions of Asn (13.2%), Gly (14.9%), and Tyr (13.2%), which accounted for 41.3% of the total amino acid residues (Supplementary Table 1). Analysis of the Pmaz-pearlin sequence revealed the presence of several characteristic domains of the low molecular weight protein family from the shell matrix, including four acidic rich regions (AR1, residues 41–50; AR2, residues 68–73; AR3, residues 89–91 and AR4, residues 132–139), ten conserved cysteine residues (Cys_18_, Cys_30_, Cys_36_, Cys_54_, Cys_55_, Cys_57_, Cys_66_, Cys_77_, Cys_85_, and Cys_86_) and an NG domain (N_92_-G_111_) (Fig. 2A).

The Pmaz-pearlin sequence showed 98.57% similarity to the Pearlin from *Pinctada margaritifera*, 92.14% to N14 from *Pinctada maxima*, and 70–74.4% to pearlin, N14, and N16 from *Pinctada fucata* (Supplementary Table 2). All the protein sequences contained a signal peptide, four aspartic residues (AR1-4), ten conserved Cys residues and the NG domain, however, the length differ among them, being pearlin from *P. mazatlanica*, *P. margaritifera* and N14 from *P. maxima* the longest from all the sequences analyzed, with 20 amino acid residues (Fig. 2A). Also, only N14.1 to N14.4 contained a heparin-binding motif (HB motif) composed by HKK residues.

Further bioinformatics analysis of the Pmaz-pearlin sequence indicates that the protein contains several putative PTMs such as O-glycosylation (Ser and Thr) and N-glycosylation (Tyr), as well as eleven phosphorylation sites. The motif TDDD, a phosphorylation site found in other homologs proteins, displayed a conservative substitution in Pmaz-pearlin (D89E). Also, four putative disulfide bridges Cys_30_-Cys_57_, Cys_54_-Cys_66_, Cys_55_-Cys_86_, and Cys_77_-Cys_85_ were predicted for Pmaz-pearlin (Fig. 2A).

### Phylogenetic analysis

Eleven homologs proteins were identified in other oyster species from the low weight molecular protein family. The phylogenetic analysis of those proteins including the deduced Pmaz-pearlin showed two main branches, the first branch contained two nodes with over 60% support, this branch was represented by all homologs protein from *P. fucata* (N14, N16, and pearlin), and the second branch with 100% support was represented by pearlins (*P. margaritifera* and *P. mazatlanica*), and one N14 from *P. maxima* (Fig. 2B).

### Characterization of isolated pearlin protein from the shell

**Figure 3:**
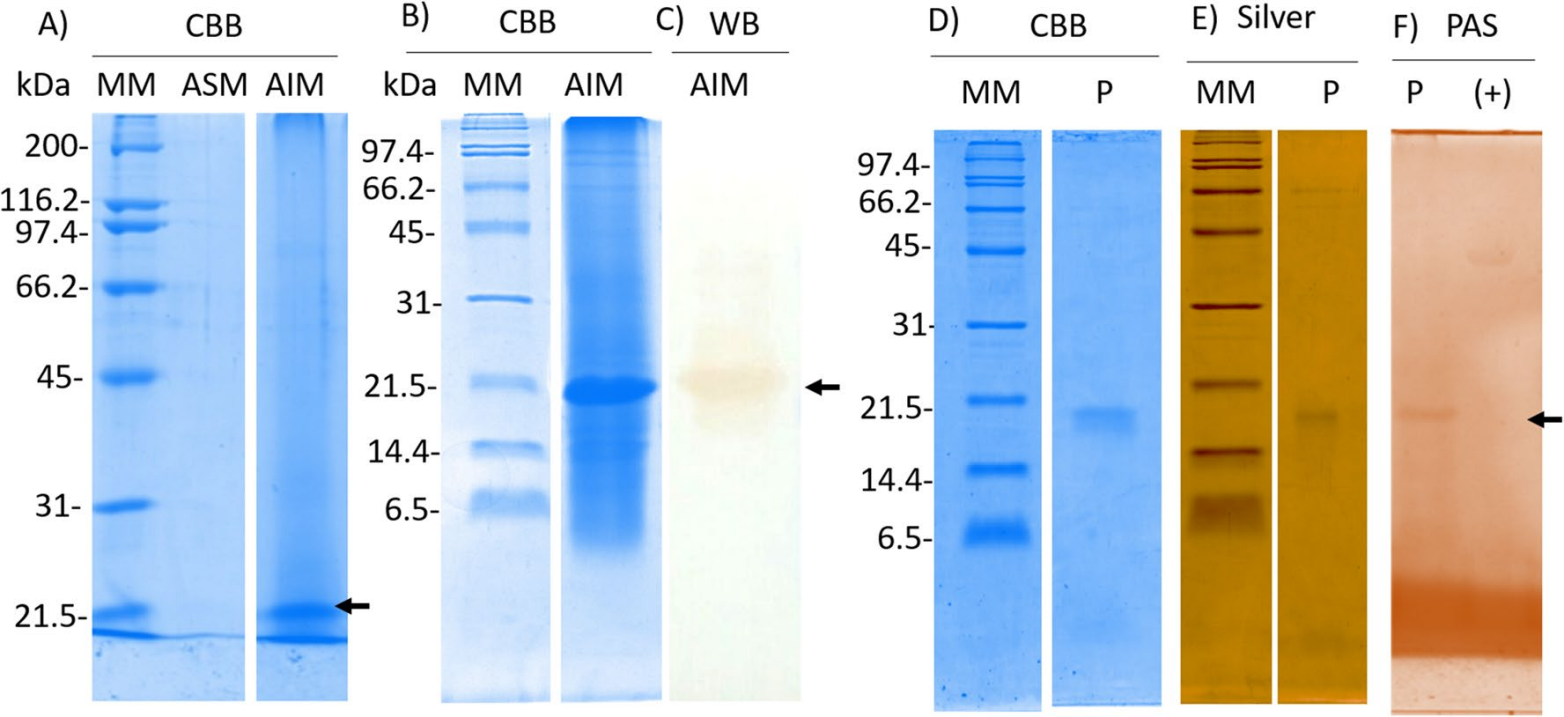
Pearlin identification and purification from the acetic insoluble matrix of the shell of *Pinctada mazatlanica*. Protein profile of the acetic soluble (ASM) and insoluble (AIM) matrix analyzed by Coomassie Brilliant Blue (**A**) 12% SDS-PAGE, and (**B**) 16% SDS-PAGE, (**C**) Immunodetection of pearlin by a polyclonal antibody against pearlin by Western blot, (**D**) Purified *P. mazatlanica* protein analyzed by 16% SDS-PAGE and stained with Coomassie Brilliant Blue, (**E**) Silver stain, and (**F**) periodic acid Schiff´s stain. MM: Molecular marker (kDa), CBB: Coomassie Brilliant Blue, PAS: periodic acid Schiff, ovalbumin protein was used as a positive control for Schiff´s stain. Gels and Blots were cropped for concise presentation, the full-length gels and blot are presented in the Supplementary Fig. 2.

Shell matrix proteins were extracted from the shell of *P. mazatlanica* using acetic acid and the acetic acid-soluble proteins (ASM) and acetic acid-insoluble proteins (AIM) were separated. The proteins from ASM and AIM were separated by 12% SDS-PAGE and only a protein band of ~ 20 kDa was identified in the AIM (Fig. 3A). This protein band (~ 20 kDa) was found to have immunoreactivity with an antibody against Pmarg-pearlin (Fig. 3B,C), suggesting that this band corresponds to a pearlin protein.

The identified pearlin, named Pmaz-pearlin, was isolated from the AIM in a single step using preparative electrophoresis. The Pmaz-pearlin was eluted in 30 fractions from 150 fractions collected. Each purification procedure produced 30.2 µg of protein from 77 µg of the target protein from the AIM, leading a yield of 38.9% (Table 1). The isolated Pmaz-pearlin has a relative molecular mass of ~ 16.74 kDa (Fig. 3D–F), which differ significantly to the deduced molecular mass, ~ 13.6 kDa, this difference could be due to the presence of PTMs. According to PAS staining of the protein on 16% SDS-PAGE, Pmaz-pearlin possesses carbohydrates associated, however, the composition of the carbohydrates was not analyzed. The Pmaz-pearlin protein band analyzed by LC–MS/MS produced four peptide sequences that matched with the deduced amino acid residues of the amplified pearlin transcript from the mantle of *P. mazatlanica* (Fig. 1, Table 2).

**Table 1:** Purification of pearlin from the shell of *Pinctada mazatlanica*.

Step	Total protein (mg)	Target protein (mg)	Yield (%)	Purity (%)
Crude extract, AIM	0.381	0.077	100.0	20.2
Preparative electrophoresis and AMICON concentrate	0.030	0.030	38.9	100.0

**Table 2:** Pearlin peptide sequences from mass spectrometry.

Peptide	Calculated mass (Da)	Observed mass (Da)
YWYCWLPYDIER	1706.94	1763.79
NAWTPWQCR	1161.30	1219.53
NAWTPWQCREDEQFER	2095.23	2151.93
YLFGGNGNGNGEFWEEYIDER	2466.56	2469.02

### In vitro crystallization of Pmaz-pearlin

**Figure 4:**
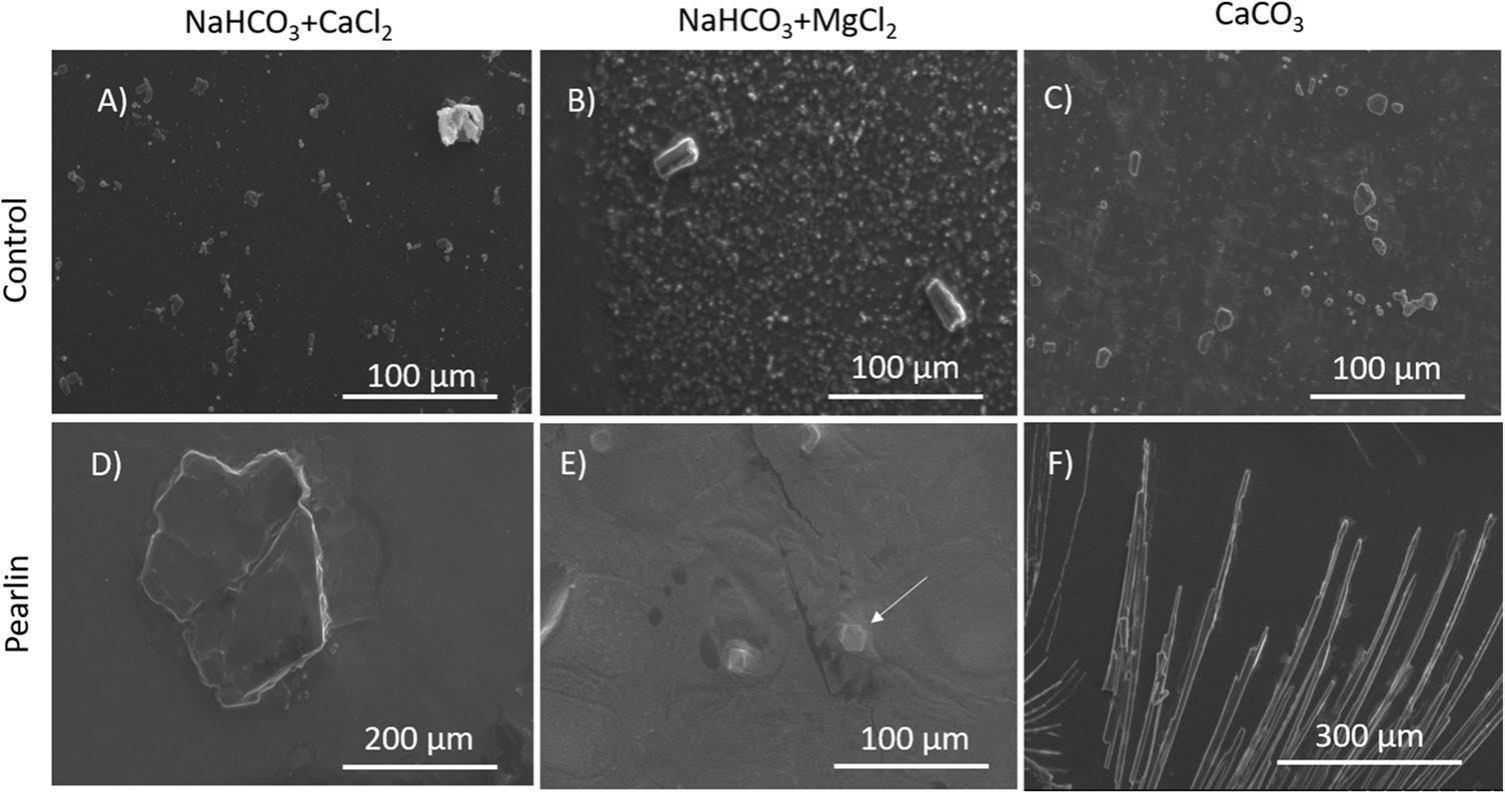
Effect of pearlin from *Pinctada mazatlanica* on CaCO_3_ crystal growth. SEM micrographs of calcium carbonate crystal growth in the presence of 10 µL of pearlin incubated at 4 °C for 21 days. Controls (**A**–**C**): Salts without pearlin; Pearlin (**D**–**F**): Salts in the presence of the isolated pearlin from the shell. Pictures are representative of three independent experiments.

Three different salts, (1) NaHCO_3_, CaCl_2_, (2) NaHCO_3_, MgCl_2_, and (3) CaCO_3_, were tested to evaluate the crystal growth in the presence of Pmaz-pearlin (Fig. 4). Control preparations, salts without protein, displayed small crystal structures of ~ 20 µm with rhombohedral structure (Fig. 4A–C). Preparations containing Pmaz-pearlin displayed structured crystals of > 100 µm, which were significantly bigger than controls. Addition of Pmaz-pearlin to the saturated solution containing CaCl_2_ formed structured crystals of 200 µm (Fig. 4D), and in the presence of MgCl_2_, nacre platelets were observed with the typical hexagonal shape (Fig. 4E). Finally, Pmaz-pearlin produced large needle-like crystals (> 300 µm) in the presence of CaCO_3_ (Fig. 4F).

### Raman analysis

**Figure 5:**
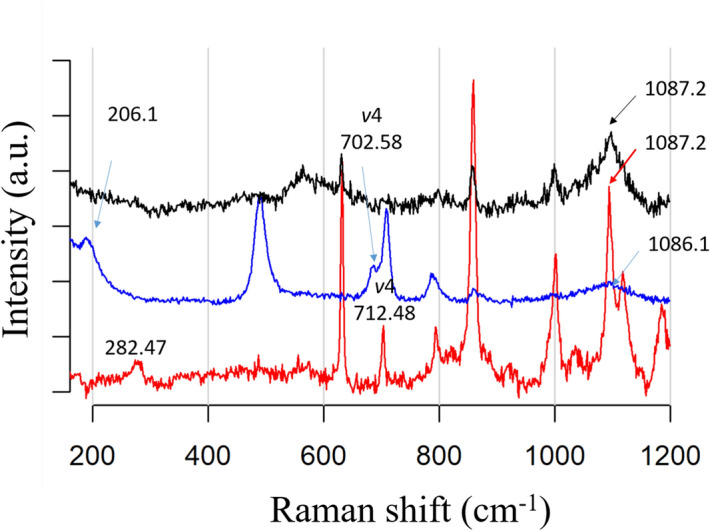
Raman spectra of calcite and aragonite crystals induced by the Pmaz-pearlin isolated from the acetic acid-soluble matrix from *Pinctada mazatlanica* shell. Red line: Calcite crystals formed in presence of CaCl_2_ and NaHCO_3_; blue line: Aragonite crystals formed in presence of MgCl_2_ and NaHCO_3_; black line: Raman spectra of crystals formed in presence of CaCO_3_.

The identity of the in vitro crystals produced by the pearlin from *P. mazatlanica* was confirmed by Raman spectroscopy. The spectra for the samples corresponding to Fig. 4D–F. are shown in Fig. 5. Theoretical calculation of calcite and aragonite crystals using Raman spectroscopy have been widely studied^31^. Calcite crystals contains two CaCO_3_ units, for a total of ten atoms, and at Raman spectrum displays translator oscillations of CO_3_ groups at 282.47 cm^−1^, asymmetric bending at 712.48 cm^−1^ (*v*4), and symmetric stretching of CO_3_ groups (*v*1), confirming the calcite structure with space group ${D}_{3d}^{6}(R3C)$ and parameters of a = 5.03 and c = 17.325 Å, respectively^32^. Aragonite crystal, contains four CaCO_3_ units, for a total of twenty atoms, and displays a Raman spectrum at 206.1 cm^−1^, 702.595 cm^−1^, and 1085.16 cm^−1^ with space group ${D}_{2h}^{16}\left(Pnma\right)$ cell parameters of 5.008, 8.029, and 5.861 Å for the a, b, and c axes, respectively^33^.

## Discussion

The nacreous layer of pearl oysters is one of the major biominerals of biotechnological interest due to its fracture toughness^34^. In bivalve mollusks, the nacre layer consists of a brick-and-mortar structure where aragonite platelets are arranged in parallel layers^35^. Their construction is performed by specific shell matrix proteins (SMPs), which are known to be secreted from the edge region and the inner part of the mantle, also known as mantle edge and pallium, respectively^36^. Once released to the extrapallial space, they form a framework that promotes the nucleation and deposition of aragonite platelets^37–40^.

Several proteins have been recognized to be involved in nacre growth, they are classified by their domain composition, such as Pif, CA, VWA, LamG-, CBD2-, Kunitz-like, WAP, M-rich, D-rich, G-rich, Q-rich, A-rich, KU-like, chitin-binding domain, or RLCD proteins^41–44^. Pearlins from *P. margaritifera* and *P. fucata*, are G-rich protein, which contains in their modular structure acidic regions, an NG domain, and Cys conserved residues which form disulfide bridges, including in *P. mazatlanica* in this study. The acidic rich regions, including the NG repeat domain, are hypothesized to induce secondary structures such as β-sheets, helices and random coils which are known to react favorably with mineral surfaces^4,45^, such as Ca^2+^ or other mineral surfaces^46^. Also, the length of the NG domain is associated with a stronger reaction with Ca^2+^ molecules, matrix components, and crystals^4^. From all pearlins and homolog proteins analyzed, only pearlin from *P. margaritifera* and *P. mazatlanica* and the N14 from *P. maxima* have a 20 amino acid residues length of the NG repeat domain (Fig. 2A), which is two times longer to N14 and N16 proteins, suggesting that these proteins might present a better capability to form calcium carbonate crystals.

Another important feature of SMPs is the presence of cysteine residues, which suggests that they form disulfide bonds. According to the bioinformatics analysis of the deduced protein sequence of Pmaz-pearlin, four putative disulfide bridges are formed, which seems to be conserved in all sequences analyzed (Fig. 2A). Several SMPs, soluble and insoluble, have disulfide bonds, such as P20^47^, P60^48^, PPP-10^49^, lustrin A^50^ and P14^51^, some of this disulfide bridges contribute to stabilize intermolecular subunits, such as P20 and P60 proteins^47,48^. The presence of disulfide bonds in SMPs, including Pmaz-pearlin, might contribute to protect the protein from degradations during the synthesis of the aragonite platelets^47,48^, as well as to provide rigidity to the structure^52^, which might impact on their function.

Moreover, posttranslational modifications have been found to be extensive in SMPs and are thought to be crucial for activity^53^. For example, glycosylation may be important for binding Ca^2+^^3^ as well as surface recognition^54^ and polymorph selection^55^; phosphorylation has been shown to be important for regulating crystal growth and sulfates are thought to bind Ca^2+^ ions and facilitate crystal nucleation^56^. Deduced pearlin sequence of *P. mazatlanica* Pmaz-pearlin possess putative O- and N-glycosylations, which was corroborated by PAS staining, and potentially have similar effects than those previously described in other SMPs^24^, however, the type of carbohydrate was not assessed, as well as the putative phosphorylation.

In vitro crystallization assay is the only assay to evaluate the activity of most of SMPs. Crystal carbonate polymorphs start with the growth of an amorphous crystal carbonate (ACC), a metastable hydrated phase, which then dehydrates to form calcite, aragonite or vaterite^57^. It is known that the presence of ions such as Mg^2+^ or Sr^2+^ or even acidic residues such as aspartic acid or citric acid, affect the crystal polymorph^58^. Magnesium ion is the most abundant ion in the shell, representing 2.55% of the total shell weight^59^ and it is known to promote aragonite crystals^60^. Pmaz-pearlin in presence of MgCl_2_ was able to produce aragonite platelets, with classic hexagonal shape, and calcite crystals in presence of CaCl_2_, which were similar to published morphological studies of aragonite and calcite by SEM^61,62^ and previous identified crystals structure by Raman analysis^31,33^, as well as those previously reported pearlins^21,24,29^. However, Pmaz-pearlin in presence of CaCO_3_ lead to needle-like crystals of aragonite, this behavior was also described for *Hyriopsis cumingii* when the whole extract was used for crystallization assay^63^. The strong capability to produce aragonite platelets and needle-like crystals of aragonite might be enhanced by the intrinsic properties of the sequences (acidic regions and NG domain) and it’s PTMs (glycosylations).

## Conclusions

The characterization of Pmaz-pearlin, contributes to the knowledge of the relatively small group of low molecular proteins involved in nacre growth. Pearlin from *P. mazatlanica* is a monomeric glycoprotein of ~ 16.74 kDa, which possesses the modular structure of the pearlins (AR, an NG domain, and disulfide bridges). The intrinsic properties of the sequences and the presence of PTMs might contribute to the capability to form aragonite platelets in the presence of MgCl_2_ and CaCO_3_.

## Methods

### Biological material

Mother pearl oysters, *Pinctada mazatlanica*, were provided by Perlas del Cortez S. de R.L. M.I. located at Bahia de la Paz B.C.S. Organisms were transported to the Molecular Genetics Laboratory at CIBNOR, and mantle tissue was dissected and stored at − 80 °C until used.

### Molecular characterization of Pearlin from mantle tissue of *P. mazatlanica*

#### Total RNA extraction and RNA reverse transcription

Total RNA was extracted with TRIzol reagent (Invitrogen Life Technologies) according to the manufacturer´s instructions. Samples (100 mg) were homogenized using a glass pestle and two consecutive extractions of each sample were made. RNA concentration and purity were determined by spectrophotometry using a NanoDrop ND-2000 at 260/280 and 260/230 nm absorbance ratios (range, 1.9–2.0). The RNA integrity was assessed on a 1% (w/v) Synergel agarose gel. To ensure complete DNA absence, a direct PCR was performed using 1 µL (50 ng µL^−1^) of each RNA preparation with 28S ribosomal (GenBank accession No. AY632555) specific primers, 28S_F (5′-GCAGGAAAAGAAACTAAC-3′) and 28S_R (5′-CCTCTAAGTGGTTTCAC-3′), a non-amplified control. After that, 1 µg of total RNA was used from each verified RNA sample for cDNA synthesis using the cloned AMV First-Strand cDNA Synthesis Reaction and oligo-dT primer. The cDNAs were stored at -80 °C until use. Control reactions were performed without template or non-reverse transcribed RNA to determine the presence of DNA^64^.

### Pearlin cDNA sequencing

A set of primers that hybridize a conserved region of the homologous pearlin nucleotide sequence (*Pinctada margaritifera*, DQ665305; *Pinctada fucata*, AB020779; *Pinctada fucata*, AB023248-AB023253; *Pinctada fucata*, AB023067, and *Pinctada maxima*, AB032612) were designed. A 414-bp region of the mother pearl oyster pearlin cDNA was amplified by PCR. The reaction included 6 µL (GoTaq Green Master Mix, Promega), 1 µL (10 µM each) of each primer 80F (5′-CATGAMGTGCACACTTCGTT-3) and 482R (5-CCGYTCRTCGATRTACTC-3), 50 ng of *P. mazatlanica* cDNA, and water to a final volume of 12 µL. PCR amplification was carried out for 5 min at 94 °C, followed by 5 cycles consisting 1 min at 94 °C, 1 min at 94 °C, 1 min at 50 °C, and 2 min at 72 °C. In the last cycle, the extension step at 72 °C lasted 10 min. PCR products were analyzed on 1% (w/v) Synergel agarose gels and visualized under UV light after staining with UView loading dye (Bio-Rad). The single band that was produced was sequenced in both directions, using the same set of primers.

The full-length sequence of pearl oyster pearlin transcript was obtained using a cDNA amplification kit (SMART RACE, Clontech Laboratories, Mountain View, CA). First-strand cDNA synthesis of 3´- and 5´-RACE was performed separately from 1 µg poly A + pearl oyster RNA obtained with a polyA Spin mRNA isolation kit (NEB England BioLabs, S1560). PCR amplification of both pearlin cDNA ends was performed using Universal primers included in the SMART Race kit and the specific primers 80F and 482R, using the 3´RACE and 5´RACE cDNA, respectively as template. Both PCR reactions produced a single PCR band that was used for ligation in a sequencing vector (pGEM-Teasy, Promega) and then cloned into *Escherichia coli* TOP10 cells (C4040, Thermo Fischer) following standard cloning methods. Plasmid DNA was isolated from three positive colonies using the alkaline lysis method and used for sequencing reactions.

### In silico analyses

Sequence similarity searches were performed using the alignment tool BLAST^65^. Pearlin homologs proteins were obtained from the National Center of Biotechnology Information (NCBI). An analysis degree of similarity among nucleotide sequences was performed using the ClustalW tool^66^. A phylogenetic using pearlin homologs proteins were constructed using MEGA software version 6.0^67^, with a bootstrapping value of 1000.

The identification of the open reading frame was performed using the ORF finder software (https://www.ncbi.nlm.nih.gov/orffinder/). Protein sequence alignment was performed with CLUSTAL Omega^66^. Identification of putative protein motifs was performed using the MotifScan (Pfam HMMs global models´ database) and SMART^68^ available at the Swiss Institute of Bioinformatics (https://myhits.sib.swiss/cgi-bin/motif_scan). Identification of signal peptide was achieved by using SignalP 4.1 Server^69^. Theoretical molecular weight, isoelectric point (pI), and amino acid composition of the protein were calculated using the ProtParam software from ExPASy (Expert Protein Analysis System; https://www.expasy.org/). Putative glycosylation and phosphorylation sites of pearlin were determined using NetOGly 4.0^70^, NetNGlyc 1.0^71^, and NetPhos2.0^72^. Also, putative disulfide bridges were determined using DiANNA 1.1 server^73^.

### Native pearlin purification and characterization

#### Shell matrix extraction

The organic matrix of prismatic and nacreous layers of *Pinctada mazatlanica* were crushed to a fine powder. The powdered matrices (20 g) were suspended in 100 mL cold acetic acid (4 °C, 10% v/v) and incubated 24 h with continuous stirring. Acetic acid-soluble matrix fractions (ASM) and acetic acid-insoluble matrix fractions (AIM) were separated by centrifugation at 13,000 × *g* for 20 min^74^. The AIM was rinsed with distilled water and lyophilized. The ASM was dialyzed for another 24 h against cold acetic acid (4 °C, 1% v/v), afterward, the ASM was dialyzed for another 24 h against distilled water, and then lyophilized.

### Sodium dodecyl sulfate–polyacrylamide gel electrophoresis

Sodium dodecyl sulfate–polyacrylamide gel electrophoresis (SDS-PAGE) was performed according to Laemmli^75^. One milligram of lyophilized ASM and AIM of the shell from *P. mazatlanica* were mixed with 50 µL of 4 × sample buffer (05 M Tris–HCl pH 6.8, 20% glycerol, 10% SDS, 10% β-mercaptoethanol and 0.05% bromophenol blue) respectively. Samples were boiled for 10 min and then centrifuged 1 min at 12,000 × *g*. The supernatant (20 µL) was loaded into a 12% polyacrylamide gel. Broad range molecular weight standard (Bio-Rad, 1610317, California, USA) was loaded into the gel. Electrophoresis was run at 90-V at room temperature, using a vertical electrophoresis unit (Bio-Rad Protean II, California, USA). After electrophoresis, the gels were stained for 2 h with staining solution (0.05% (w/v) Coomassie Brilliant Blue R-250, 7% (v/v) acetic acid, 40% (v/v) methanol). Proteins were revealed by soaking for 2 h in distaining solution (7% (v/v) acetic acid and 40% (v/v) methanol). Gel proteins were analyzed using a gel imager (Chemi Doc XRS, Bio-Rad, California, USA).

### Protein quantification

Quantification of pearlin present in the AIM was performed by pixel densitometry as described by Arroyo-Loranca^74^. Briefly, a standard curve was performed using ovalbumin protein (0.25–7.0 µg/µL) and the linear equation obtained was used for the pearlin protein quantification, y = 10138x-4163.7 (Supplementary Fig. 1).

### Immunodetection of pearlin by Western Blot

Polyclonal antibodies against recombinant pearlin from *Pinctada margaritifera*^24^ were used to detect the pearlin of the ASM and AIM from the shell of *Pinctada mazatlanica*. Protein samples (1 mg of lyophilized ASM and AIM) were separated using a 12% electrophoresis gel under reducing conditions as described before. Afterward, the proteins were electrophoretically transferred to a PVDF membrane with a semidry blotter (Thermo Fischer Scientific) at 18 V, 90 mA for 30 min. Then, the PVDF membrane was blocked with 5% skimmed milk in TNT buffer (10 mM Tris–HCl pH 8.0, 0.15 M NaCl, 0.05% Tween-20), incubated with the primary polyclonal antibody against pearlin (1:10,000), and followed with the second antibody anti-goat horseradish peroxidase conjugate (Santa Cruz, USA) at a 1:5000 dilution. Finally, the immunoblots were visualized in 3,3′-diaminobenzidine (DAB) solution.

### Pearlin purification by preparative SDS-PAGE

The identified pearlin from the AIM was isolated using a preparative polyacrylamide gel electrophoresis according to the Mini-Prep Cell Manual (Bio-Rad, model 491 Prep Cell), using 20 mg of lyophilized AIM according to Arroyo-Loranca^74^. Proteins containing the pearlin protein were pooled, concentrated using an Amicon-Ultra filter (10,000 MW cut-off) and desalted using 30 mM Tris–HCl buffer pH 8.8. Isolated protein was stored at − 20 °C until use.

### Mass spectrometry analysis

Pearlin protein (2 µg) was separated by 12% SDS-PAGE, stained with Coomassie Brilliant Blue, and the band manually cut and extracted for the gel. The gel band was digested with trypsin and processed at the Laboratorio Universitario de Proteómica, UNAM.

### Posttranslational modification analysis

Putative glycosylation of pearlin (2 µg) was determined by 12% SDS-PAGE polyacrylamide gels. Saccharide moieties were identified with Periodic Acid-Schiff Stain (PAS) (Sigma-Aldrich, S5133, St. Louis MO, USA)^76^.

### Crystallization in vitro assay of pearlin

Growth of calcium carbonate crystals (calcite and/or aragonite) in the presence of purified pearlin was performed in vitro. Three different salt solutions were tested according to Weiss^77^ and Hillner^78^, solution A containing 40 mM CaCl_2_ pH 8.2, 100 mM NaHCO_3_, solution B containing 40 mM MgCl_2_ pH 8.2, 100 mM NaHCO_3_, and solution C containing 100 mM CaCO_3_. All solutions were prepared using molecular biology grade reagents. Two micrograms of protein (10 µL) were mixed with 35 µl of each solution respectively, controls without protein were included. Each mixture was incubated over a sterile coverslip inside a six-well microplate and sealed with parafilm at 4 °C for 30 days. Each experiment was performed by triplicate. The morphology of the crystal was determined by Scanning Electron Microscopy (SEM) by triplicate at the Electronic Microscopy Laboratory at Centro de Investigaciones Biológicas del Noroeste S.C. (CIBNOR), México.

### Raman

The identity of the in vitro crystals was obtained using Raman spectroscopy using an InVia microRaman spectrometer, using a 532 nm laser (YAG laser) as the excitation source at 100 mW power with a spot size of 4 µm^2^. The crystals were scanned from 100 to 1200 by triplicate for the specific identification. For all the measurements, the slits were set at 200 µm and a 100 × objective was used. The analysis was carried out at Laboratorio Nacional de Investigaciones en Nanociencias y Nanotecnologia (LINAN)-IPICyT.

## Supplementary information

Supplementary information.
