# Supplementary material for: A shell matrix protein of Pinctada mazatlanica produces nacre platelets in vitro
Source: Sci Rep. 2020 Nov 19;10:20201. doi: 10.1038/s41598-020-77320-7 (PMC7677314; doi:10.1038/s41598-020-77320-7)
Supplement: Supplementary file 1 — Supplementary information. [file 41598_2020_77320_MOESM1_ESM.docx]

**A shell matrix protein of *Pinctada mazatlanica* produces nacre platelets in vitro**

Crisalejandra Rivera-Perez^1^, Iliana Alejandra Flores Sánchez^2^, Josafat Jehu Ojeda-Ramírez de Areyano^3^, Delia Irene Rojas Posadas^3^, Norma Y. Hernández Saavedra^3^

^1^CONACYT-Centro de Investigaciones Biológicas del Noroeste (CIBNOR), Avenida Instituto Politécnico Nacional No. 195, Playa Palo de Sta. Rita Sur, Apartado Postal 128, La Paz 23096, Baja California Sur, Mexico.

^2^Tecnológico Nacional de México, La Paz, Baja California Sur, Mexico.

^3^Centro de Investigaciones Biológicas del Noroeste (CIBNOR), Avenida Instituto Politécnico Nacional No. 195, Playa Palo de Sta. Rita Sur, Apartado Postal 128, La Paz 23096, Baja California Sur, Mexico.


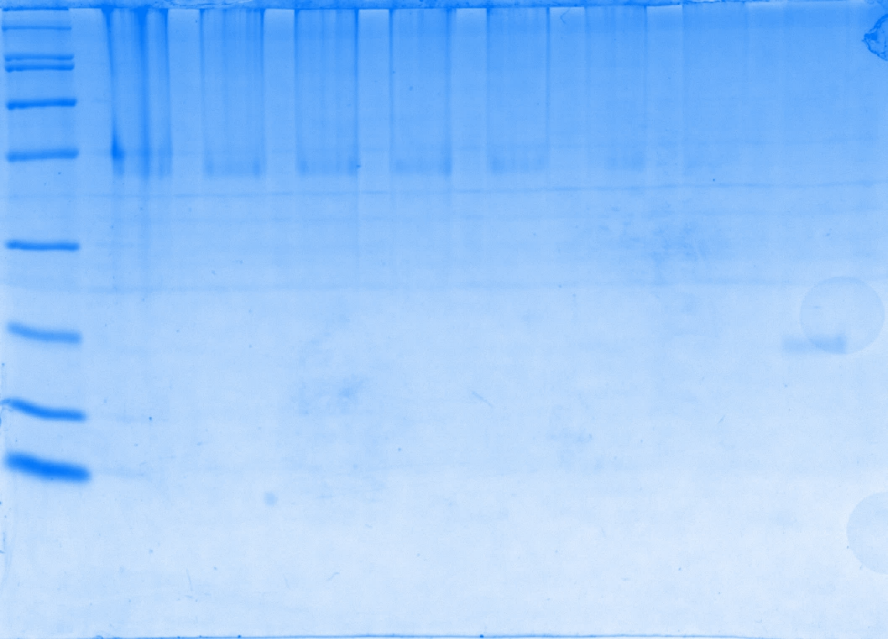


MM 8.0 7.0 5.0 3.0 1.0 0.5 0.25 S

97.4-

66.2-

45-

31-

21.5-

14.4-

6.5-

A)

B)


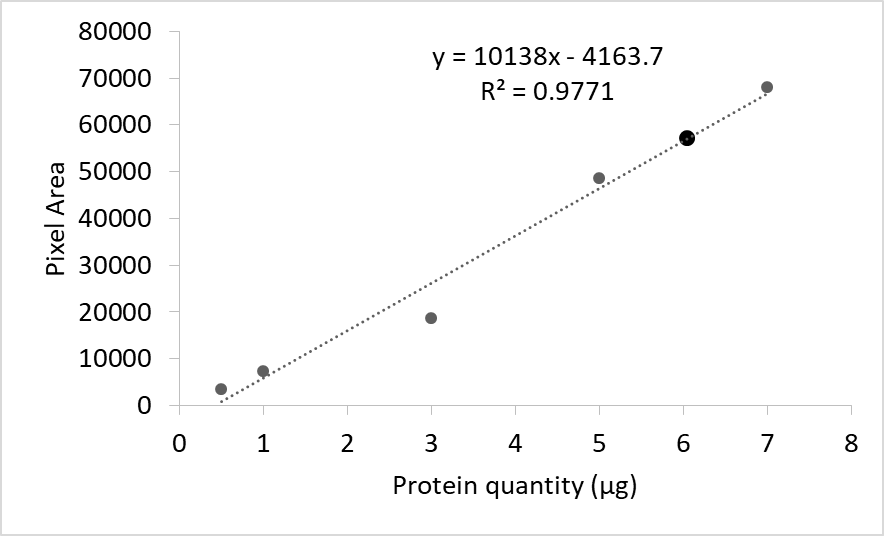


**Supplementary Figure 1. Ovalbumin standard curve.** (A) SDS-PAGE 16% polyacrylamide gel. Ovalbumin standard curve indicated by an arrow (8.0-0.25 µg/µl) stained with Coomassie Brilliant Blue for pixel density determination to calculate the linear equation and quantify protein bands. MM: molecular marker; Ovalbumin concentrations (µg/µl)*.* (B) Ovalbumin standard curve graphic. The axes represent the pixel area and protein quantity (µg). Gray circles represent Ovalbumin (µg), the filled black circle indicates the purified *Pinctada mazatlanica* pearlin.


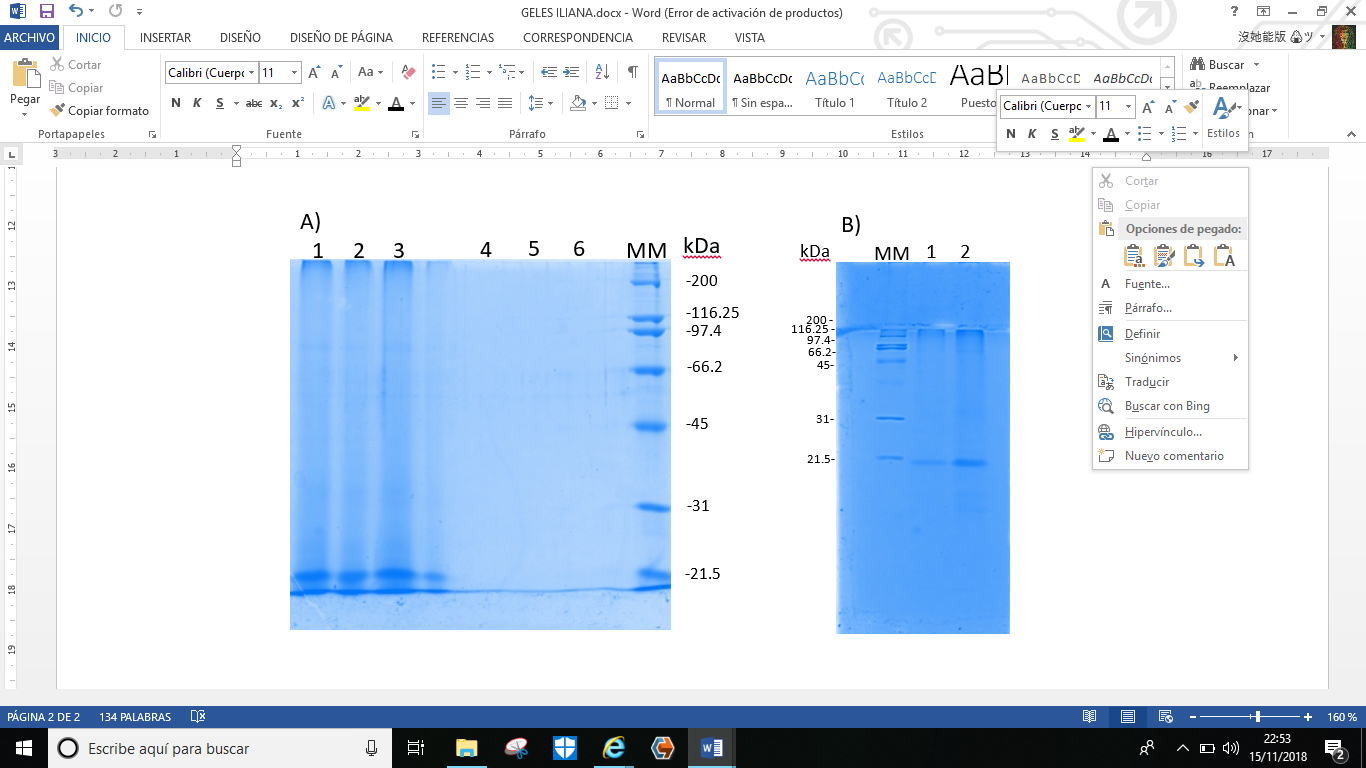


200-

116.2-

97.4-

66.2-

45-

31-

21.5-

MM ASM X X X X AIM X X

**Supplementary Figure 2.** Raw image of Figure 3A: Protein profile of the acetic soluble (ASM) and insoluble (AIM) matrix analyzed by Coomassie Brilliant Blue on 12% SDS-PAGE. MM: Molecular marker, X: denotes lines not included in the main manuscript.


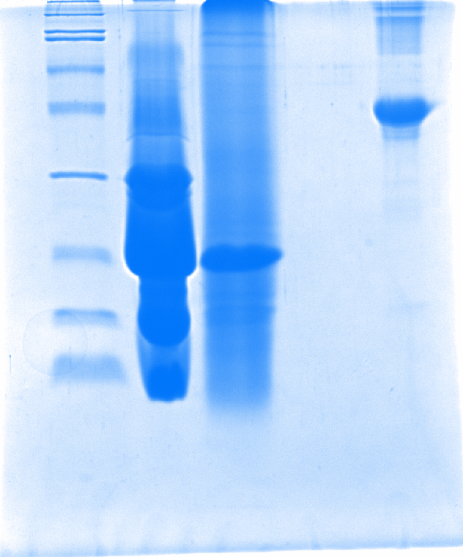


97.4-

66.2-

45-

31-

21.5-

14.4-

6.5-

MM X AIM X X

**Supplementary Figure 2.** Raw image of Figure 3B: Protein profile of the acetic insoluble (AIM) matrix analyzed by Coomassie Brilliant Blue on 16% SDS-PAGE. MM: Molecular marker, X: denotes lines not included in the main manuscript.


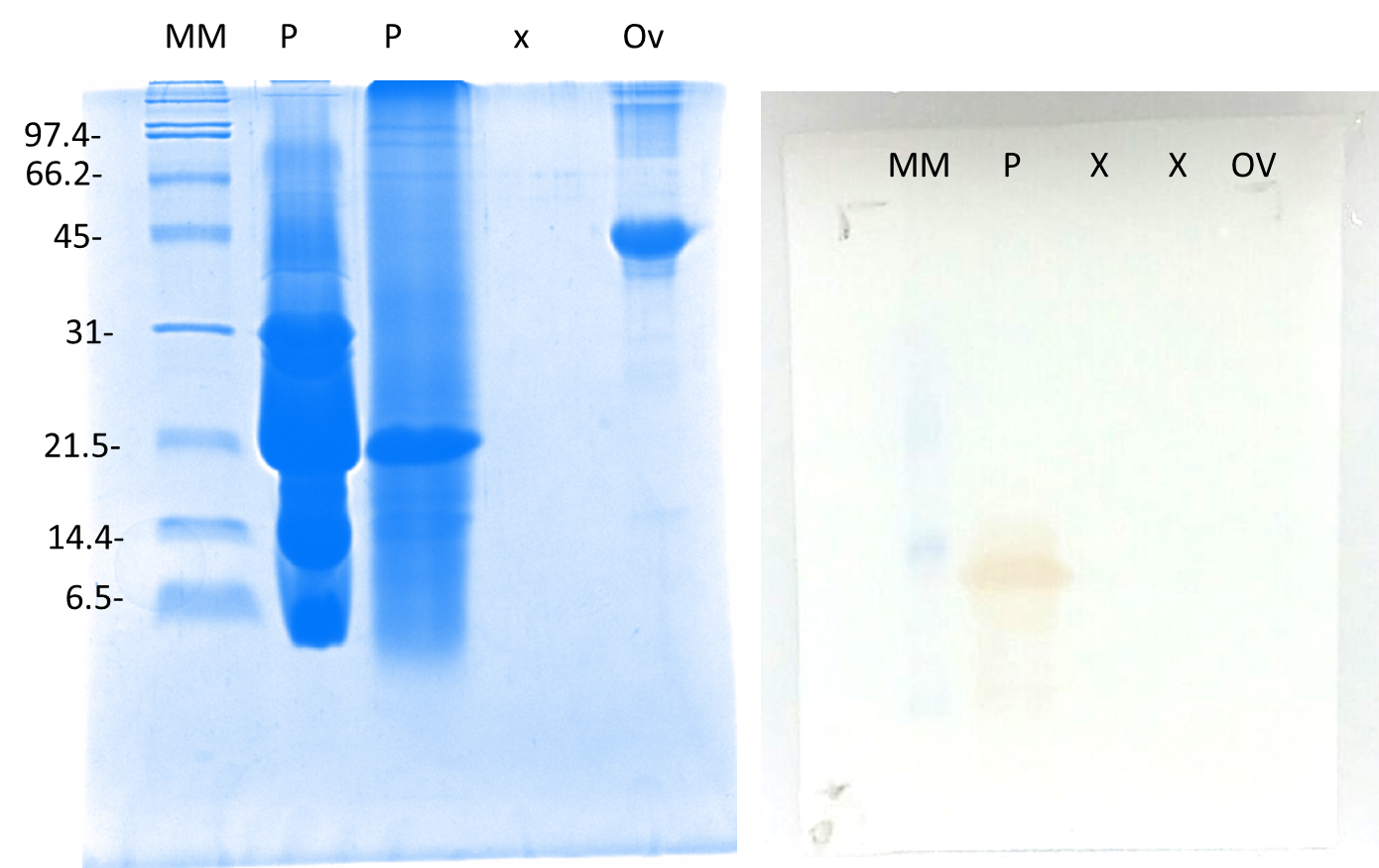


**Supplementary Figure 2.** Raw image of Figure 3C: Western Blot (right) of AIM analyzed with antibodies against pearlin from *Pinctada margaritifera.* Image on the left, the Coomassie Brilliant Blue sample run parallel of the same samples of the western blot. MM: molecular marker, P: acetic insoluble extract protein, Ov: ovalbumin. X: denotes lines not included in the figure of the manuscript.


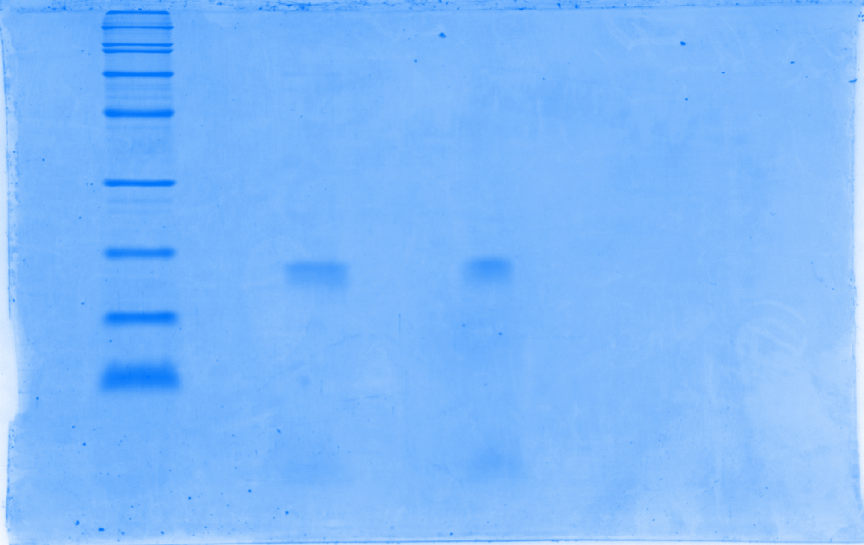


97.4-

66.2-

45-

31-

21.5-

14.4-

6.5-

MM X P X X X X X X

**Supplementary Figure 2.** Raw image of Figure 3D: Protein profile of the isolated pearlin analyzed by Coomassie Brilliant Blue on 16% SDS-PAGE. MM: Molecular marker, P: protein, X: denotes lines not included in the main manuscript.


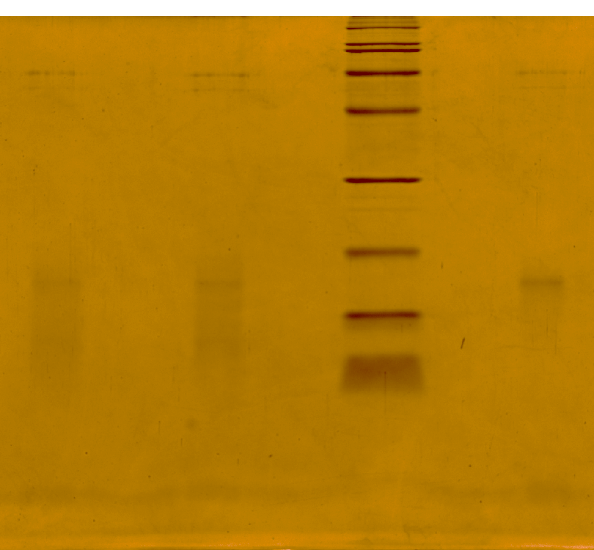


97.4-

66.2-

45-

31-

21.5-

14.4-

6.5-

X X X X MM X P

**Supplementary Figure 2.** Raw image of Figure 3E: Protein profile of the isolated pearlin analyzed by Silver stain on 16% SDS-PAGE. MM: Molecular marker, P: protein, X: denotes lines not included in the main manuscript.


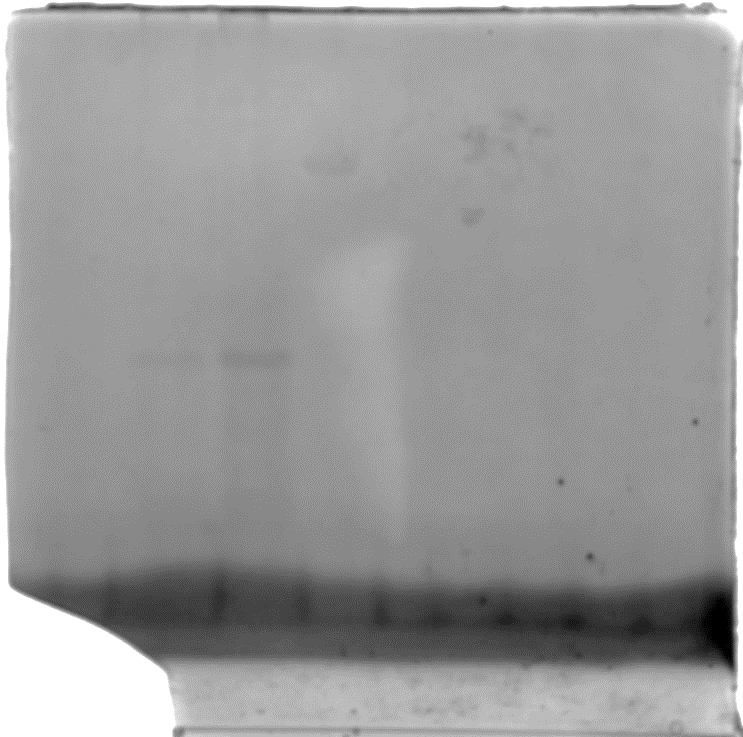


x x P O x x x x x

**Supplementary Figure 2.** Raw image of Figure 3F: Periodic Acid Schiff stain of isolated pearlin from the shell of *Pinctada mazatlanica.* P: pearlin, O: ovalbumin, X: lines not included in the manuscript.

**Supplementary Table 1.** Amino acid composition of pearlin from the shell of *P. mazatlanica*.

| **Amino acid residue** | **Residues** | **Mol%** |
| --- | --- | --- |
| Ala | 6 | 4.3% |
| Arg | 12 | 8.6% |
| Asn | 15 | 10.7% |
| Asp | 9 | 6.4% |
| Cys | 10 | 7.1% |
| Gln | 3 | 2.1% |
| Glu | 9 | 6.4% |
| Gly | 18 | 12.9% |
| His | 1 | 0.7% |
| Ile | 3 | 2.1% |
| Leu | 12 | 8.6% |
| Lys | 3 | 2.1% |
| Met | 1 | 0.7% |
| Phe | 3 | 2.1% |
| Pro | 4 | 2.9% |
| Ser | 2 | 1.4% |
| Thr | 5 | 3.6% |
| Trp | 5 | 3.6% |
| Tyr | 15 | 10.7% |
| Val | 4 | 2.9% |

**Supplementary Table 2.** Percent identity matrix of selected pearlin and homolog sequences.
